# A *Plasmodium* Calcium-Dependent Protein Kinase Controls Zygote Development and Transmission by Translationally Activating Repressed mRNAs

**DOI:** 10.1016/j.chom.2012.05.014

**Published:** 2012-07-19

**Authors:** Sarah Sebastian, Mathieu Brochet, Mark O. Collins, Frank Schwach, Matthew L. Jones, David Goulding, Julian C. Rayner, Jyoti S. Choudhary, Oliver Billker

**Affiliations:** 1 Wellcome Trust Sanger Institute, Hinxton, Cambridge CB10 1SA, UK

## Abstract

Calcium-dependent protein kinases (CDPKs) play key regulatory roles in the life cycle of the malaria parasite, but in many cases their precise molecular functions are unknown. Using the rodent malaria parasite *Plasmodium berghei,* we show that CDPK1, which is known to be essential in the asexual blood stage of the parasite, is expressed in all life stages and is indispensable during the sexual mosquito life-cycle stages. Knockdown of CDPK1 in sexual stages resulted in developmentally arrested parasites and prevented mosquito transmission, and these effects were independent of the previously proposed function for CDPK1 in regulating parasite motility. In-depth translational and transcriptional profiling of arrested parasites revealed that CDPK1 translationally activates mRNA species in the developing zygote that in macrogametes remain repressed via their 3′ and 5′UTRs. These findings indicate that CDPK1 is a multifunctional protein that translationally regulates mRNAs to ensure timely and stage-specific protein expression.

## Highlights

► CDPK1 is an abundant kinase of invasive and noninvasive life-cycle stages ► It functions in gametocyte emergence and zygote-to-ookinete transformation ► CDPK1 mediates transcriptional activation of some silenced mRNAs in the zygote ► CDPK1 is crucial for the mosquito transmission of *P. berghei*

## Introduction

In malaria parasites, intracellular Ca^2+^ signals are translated into stage-specific cellular responses by members of a family of Ca^2+^-dependent protein kinases (CDPKs), which combine an N-terminal kinase domain with a C-terminal, calmodulin-like regulatory domain in the same polypeptide (Billker et al., 2009). This domain architecture is limited to alveolates and plants and results in a unique activation mechanism distinct from that of related human calmodulin dependent protein kinases (Ojo et al., 2010; Wernimont et al., 2010). Reverse genetics experiments have revealed essential functions in male gametogenesis and mosquito transmission of *P. berghei* for CDPK4 (Billker et al., 2004) and in schizont egress of *P. falciparum* for CDPK5 (Dvorin et al., 2010). The function of CDPK1, in contrast, has remained poorly understood, despite it being the recent target of different drug development efforts (Kato et al., 2008; Lemercier et al., 2009). The *cdpk1* gene is likely to be essential in asexual blood stages, since it has resisted disruption in *P. falciparum* (Kato et al., 2008) and *P. berghei* (Tewari et al., 2010). Transcription of the *cdpk1* gene parallels that of genes encoding the molecular motor that powers red blood cell invasion by merozoites. This, and the ability of recombinant CDPK1 to phosphorylate proteins of the motor complex in vitro, gave rise to the hypothesis that CDPK1 may be a key regulator of the motor (Green et al., 2008; Kato et al., 2008). Consistent with this idea, CDPK1 localizes to the inner layer of the plasmalemma in merozoites as a result of dual acylation of its N terminus (Green et al., 2008; Möskes et al., 2004). However, neither in vivo substrates nor genetic data are available to support the motor hypothesis.

We here examine functions of CDPK1—and those of its putative substrates in the motor complex–through stage-specific gene knockdown in sexual stages and ookinetes of *P. berghei*, a malaria parasite infecting rodents. When taken up in a mosquito blood meal, *Plasmodium* transmission stages, the gametocytes, rapidly differentiate into gametes. After gamete fusion and fertilization, the zygote undergoes meiosis and begins a major transformation into a motile ookinete, which is completed within 24 hr. Many of the proteins that distinguish the ookinete from the preceding gamete stages are translated from presynthesized messenger RNAs (mRNAs) that remain translationally repressed in the female gametocyte. These mRNAs are stabilized through association with a messenger ribonucleoprotein (mRNP) complex that includes the RNA helicase DOZI (Development of Zygote Inhibited) and the Sm-like factor CITH (homolog of worm CAR-I and fly Trailer Hitch) (Mair et al., 2006, 2010). Among the translationally controlled genes are those encoding the major ookinete surface proteins, p28 and p25, constituents of the molecular motor and inner membrane complex, and proteins targeted to the micronemes (Mair et al., 2010), secretory organelles required for gliding and invasion. In *P. berghei*, mRNAs for p28 and p25 become activated before fertilization, upon macrogametocyte activation (Paton et al., 1993), whereas the majority of translationally controlled proteins are made only after fertilization, in the differentiating zygote and ookinete. The mechanisms underlying the staged translational activation of different mRNAs are not understood, but likely require regulation by protein kinases.

Once fully developed, the ookinete leaves the hostile environment of the digesting blood meal and penetrates the midgut wall. As in other apicomplexan zoites, ookinete motility is powered by an actomyosin motor located within the parasite's pellicle. In tachyzoites of *Toxoplasma gondii*, motility and invasion require a class XIV myosin, MyoA (Meissner et al., 2002), which is anchored to the inner membrane complex (IMC), a system of flattened membrane-bound vesicles underlying most of the plasmalemma of apicomplexan invasive stages. MyoA acts on short actin filaments, which are linked to stage-specific transmembrane adhesins via aldolase. The resulting posterior translocation of the adhesin generates the power for gliding and invasion by the parasite. MyoA is associated with a light chain, which in *Plasmodium* is referred to as MyoA tail domain interacting protein, MTIP. Two gliding associated proteins, GAP45 and GAP50, anchor the MTIP-MyoA complex to the IMC (Frénal et al., 2010). In *T. gondii*, GAP45 is associated with both the PM and IMC, stretching across the supra-alveolar space (Frénal et al., 2010). The C-terminal region of GAP45 also interacts with the transmembrane protein GAP50, thereby anchoring the motor complex in the IMC. The major elements of the motor are thought to be functionally conserved in all invasive stages of malaria parasites (Baum et al., 2006; Green et al., 2006), and it is presumed that the complex is an active regulation point in all apicomplexan species. Several proteins of the *Plasmodium* motor complex are phosphorylated (Green et al., 2008; Treeck et al., 2011). Candidate kinases that could be responsible include Protein Kinase B and the CDPKs. In ookinetes, motility requires CDPK3 and cGMP dependent signaling, most likely through cyclic GMP-dependent protein kinase (Moon et al., 2009), but a role for CDPK1 has not been investigated.

We here show that gametocytes, ookinetes, and other life cycle stages of *P. berghei* express CDPK1. We demonstrate that CDPK1 is essential for the transmission of *P. berghei* to the mosquito and for multiple aspects of sexual development, including egress of male gametes from activated gametocytes, and for differentiation of the zygote into an ookinete. We examine the role of CDPK1 in regulating motor complex activity, but find that any such a role is not sufficient to explain the developmental arrest seen in the CDPK1-depleted zygotes. However, evidence from quantitative transcriptome and proteome profiling suggests a crucial role for CDPK1 upstream of the translational activation of stored mRNAs, a process required for the formation of functional ookinetes. This unexpected function for CDPK1 broadens our understanding of the regulatory role that this class of kinases plays during *Plasmodium* development.

## Results

### CDPK1 Is Expressed at Many Life-Cycle Stages

**Figure 1:**
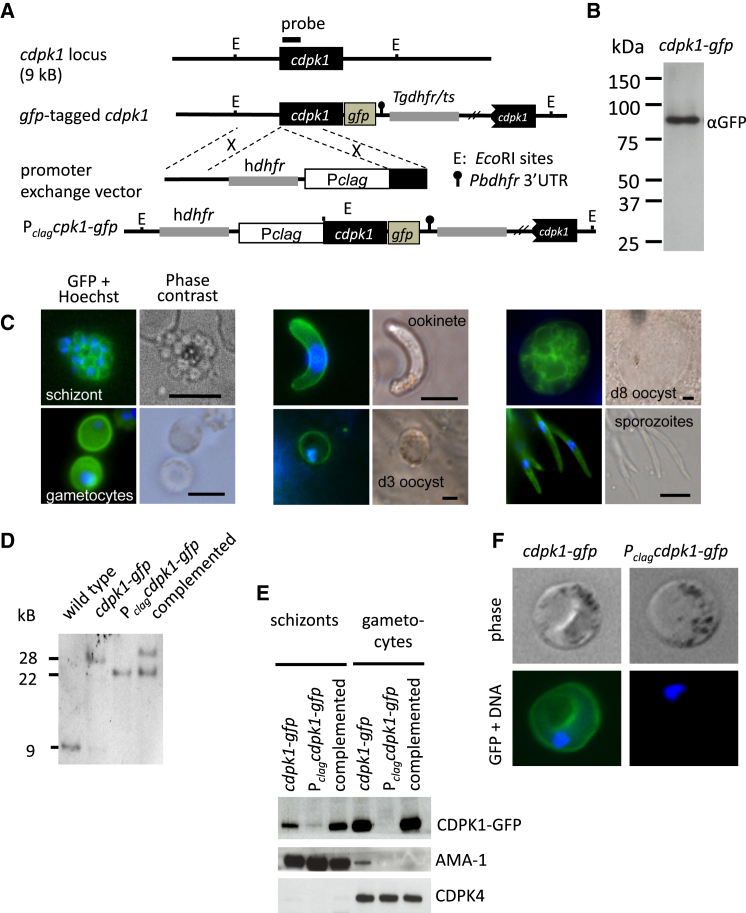
GFP Tagging and Promoter Exchange at the Endogenous *cdpk1* Locus (A) Schematic showing the *cdpk1* locus before and after insertion of a *gfp* sequence and the subsequent insertion of a *clag* promoter upstream of *cdpk1-gf*. The Southern probe and EcoRI sites (E) used for genotyping are indicated. (B) Western blot analysis of schizonts showing the fusion protein is expressed as a single protein of the expected size. (C) Fluorescence micrographs of live parasites expressing CDPK1-GFP. Each pair of panels shows an overlay of GFP fluorescence (green) and DNA labeled with Hoechst (blue) on the left and a phase contrast image on the right. (D) Southern blot analysis of EcoRI-restricted genomic DNA from transgenic clones, including one complemented with an extra copy of *cdpk1* under its endogenous promoter in the *cssu* locus. (E) Western blot analysis of equal numbers of parasites. AMA-1 and CDPK4 antibodies were used to verify equal loading of schizonts and gametocytes, respectively. (F) Impact of *clag* promoter exchange on CDPK1-GFP fluorescence in activated macrogametocytes by IFA as in (C). See also Table S3.

To study the expression and subcellular localization of CDPK1 throughout the life cycle, we generated a *P. berghei* line in which enhanced *gfp* was fused in frame with the open reading frame of the endogenous *cdpk1* gene (Figure 1A). The CDPK1-GFP fusion protein with the expected molecular weight (Figure 1B) was expressed from the endogenous *cdpk1* promoter. The C-terminal GFP tag caused no obvious growth defect in blood stages and did not interfere with parasite transmission through *Anopheles stephensi* mosquitoes. Live fluorescence microscopy showed CDPK1-GFP expression in schizonts, micro- and macrogametocytes, and all mosquito stages (Figure 1C). In schizonts, gametocytes, ookinetes, and young oocysts, much of the fluorescence signal emanated from the cell periphery suggesting dual N-terminal acylation may target the kinase to the plasmalemma, as has been demonstrated for CDPK1 in *P. falciparum* schizonts (Green et al., 2008; Möskes et al., 2004).

### Stage-Specific Knockdown of CDPK1 by Promoter Swap

The essential role of CDPK1 in asexual blood stages has so far prevented its functional analysis by reverse genetics. To overcome this limitation, we used the promoter exchange strategy of Laurentino et al. (2011) and placed the CDPK1-GFP fusion gene under the control of 1.8 kb of the 5′ upstream sequence of PBANKA_140060 (Figure 1A), a member of the *rhopH1*/*clag* multigene family, whose products are abundant in asexual stages, but absent from *P. berghei* gametocytes and ookinetes. The genotype of the resulting P_*clag*_-*cdpk1-gfp* mutant was confirmed by Southern blot (Figure 1D) and diagnostic PCR analysis (data not shown). Insertion of the *clag* 5′ sequence significantly reduced the level of CDPK1-GFP expression in schizonts (Figure 1E, lane 1 cf. lane 2), but the parasites were still viable, suggesting that the bulk of the CDPK1 protein contained in asexual blood stages is not required for growth. In gametocytes, by contrast, CDPK1-GFP expression was reduced below the detection limit of western blot (Figure 1E) and fluorescence microscopy (Figure 1F). As a control, we complemented the stage-specific expression line in *trans* by inserting a P_*cdpk1*_-*cdpk1-gfp* expression cassette into the nonessential *cssu* locus of a wild-type *P. berghei* strain, then crossing this line with the P_*clag*_-*cdpk1-gfp* mutant by feeding mosquitoes on a mouse infected with both transgenic parasites. Sporozoites originating from the cross were used to infect another mouse and parasites were cloned by limiting dilution. PCR genotyping of progeny clones identified a recombinant carrying both the P_*clag*_-*cdpk1-gfp* allele in the *cdpk1* locus and the complementing P_*cdpk1*_-*cdpk1-gfp* allele in the *cssu* locus (Figure 1D; other genotyping data not shown). Complementation in *trans* completely restored CDPK1-GFP expression levels in schizonts and gametocytes (Figure 1E) and was used as control in subsequent experiments.

### CDPK1 Is Essential for Ookinete Differentiation and Mosquito Transmission

**Figure 2:**
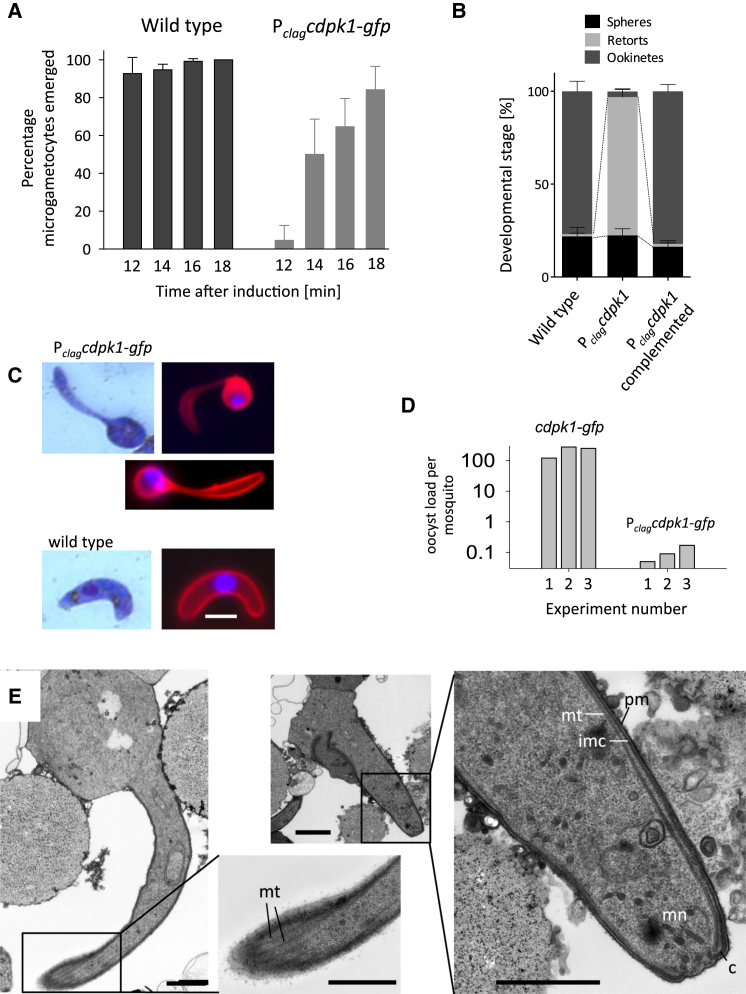
Phenotype of a P_*clag*_-*cdpk1*-*gfp* Mutant Clone (A) The proportion of exflagellating microgametocytes that had completely emerged from their host erythrocyte was scored by light microscopy at different times after the induction of gametogenesis in vitro. Error bars represent ± SD; n = 3. (B) The impact of *cdpk1-gfp* knockdown on zygote-to-ookinete conversion was assessed 24 hr after inducing gametogenesis in vitro by scoring the developmental states of 100 parasites labeled with anti-p28-Cy3 by fluorescence microscopy. Error represent ± SD; n = 3. (C) Representative images of P_*clag*_-*cdpk1*-*gfp* and wild-type ookinetes on Giemsa-stained blood films and after anti-p28-Cy3 labeling (red) of live ookinetes. DNA was stained Hoechst dye (blue) in fluorescence images. The scale bar represents 2 μm. (D) Average oocyst numbers per mosquito midgut 10 days after feeding on infected mice. Number of mosquitoes counted per parasite line: experiment 1, n = 20; experiments 2 and 3, n = 35. (E) Transmission electron micrographs of representative P_*clag*_-*cdpk1*-*gfp* ookinetes showing the  plasmalemma (pm), inner membrane complex (imc), subpellicular microtubules (mt), apical complex with micronemes (mn) and collar (c). The scale bar represents 1 μm. See also Figure S1.

We first assessed the impact of the *clag* promoter swap on the ability of gametocytes to develop into gametes in vitro when stimulated by a drop in temperature combined with the mosquito factor, xanthurenic acid. The rapid cellular differentiation of microgametocytes into eight sperm-like microgametes, a process called exflagellation, was largely unaffected by a lack of CDPK1; however, lysis of the host cell membrane(s) surrounding the microgametocyte was delayed by about 5 min. This resulted in a striking phenotype, in which flagellar movement of the forming microgametes commenced while the gametocyte was still inside the host cell (Figure 2A). P_*clag*_-*cdpk1-gfp* gametes eventually emerged, fertilized, and zygotes began to differentiate into ookinetes. While >80% of *cdpk1-gfp* macrogametocytes converted completely into morphologically mature ookinetes, differentiation of P_*clag*_-*cdpk1-gfp* zygotes was arrested at a point reminiscent of the “retort” stage of normal ookinete development (Figures 2B and 2C, and Figure S1 available online). Retorts remained immotile (Movie S1), confirming that they had not completed ookinete development. When *A. stephensi* mosquitoes were allowed to feed directly on infected mice, knockdown of CDPK1 caused a >2000-fold reduction in oocyst numbers on the midgut epithelium (Figure 2D), demonstrating an essential function for the kinase in parasite transmission to the mosquito in vivo. Complementation of the P_*clag*_-*cdpk1-gfp* mutant restored both normal microgametocyte emergence (data not shown) and ookinete development (Figure 2B), demonstrating that the experimentally induced downregulation of CDPK1 expression was responsible for these phenotypes. Transmission electron micrographs of CDPK1-deficient retort-like parasites revealed no gross abnormalities that could account for the morphology defect of the mutant, which had a completely assembled apical complex with collar, apical rings and micronemes (Figure 2E), and a typical pellicle, consisting of the plasma membrane, the inner membrane complex and regularly spaced subpellicular microtubules. CDPK1 therefore appears to regulate cellular morphogenesis in *P. berghei* ookinetes through an unknown mechanism.

### MTIP and MyoA Are Required for Ookinete Gliding but Not Morphogenesis

**Figure 3:**
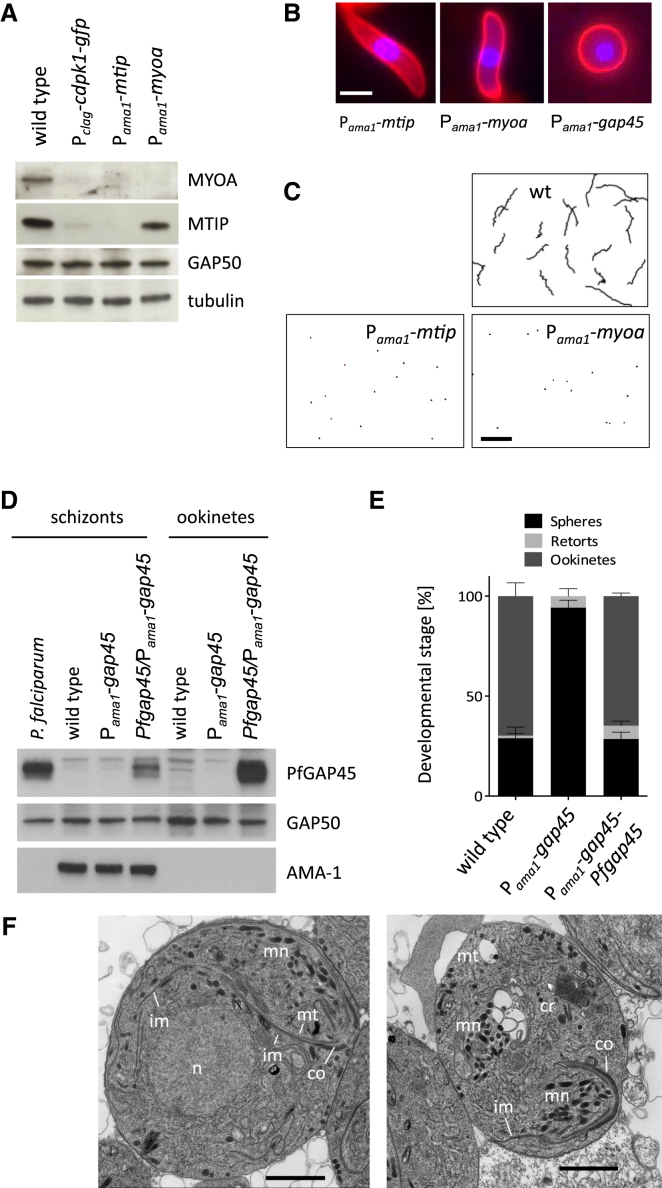
Functional Analysis of the Motor Complex in Ookinetes (A) Western blot of ookinete lysates investigating the expression of MyoA and MTIP in a panel of mutants. Tubulin and GAP50 serve as loading controls. (B) Live fluorescence images of representative zygotes/ookinetes after surface labeling with anti-p28-Cy3 (red), showing different morphology of mutants. The nuclear stain is Hoechst 33342 (blue). The scale bar represents 2 μm. (C) Motility traces of ookinetes in Matrigel were recorded during 20 min from a representative field of view. The scale bar represents 50 μm. (D) Western blot analysis of PfGAP45 expression in parasite lysates. GAP50 serves as loading control. An antibody against the AMA-1 protein shows absence of schizonts from the ookinete sample. (E) Impact of *gap45* promoter exchange on zygote-to-ookinete conversion. One hundred p28-positive parasites from 24 hr in vitro culture were scored. Error bars show standard deviations from three biological replicates. (F) Representative transmission electron micrographs of zygotes from P_*ama1*_*-gap45* mutants after 20 hr of culture. Note the intracellular apical complex with intact collar (co) and micronemes (mn). Inner membranes (im) are associated with microtubules (mt), but not with the plasmalemma. n, nucleus; cr, crystalloid. See also Figure S2 and Movie S1.

The only known in vitro substrates of CDPK1 are components of the motor complex. We therefore reasoned that components of the motor might have a function in generating and maintaining normal ookinete morphology. Consistent with this idea, MyoA and MTIP protein levels were strongly reduced in P_*clag*_-*cdpk1-gfp* retorts (Figure 3A). In contrast, expression of GAP50, which anchors the motor complex to the inner membrane complex, was not affected. A time course experiment showed MTIP levels were low at all times in P_*clag*_-*cdpk1-gfp* ookinetes, while MTIP appeared from 9 hr after fertilization in the wild-type, i.e., before manifestation of the retort phenotype in the mutant (Figure S1). The loss of MTIP from the mutant meant that we could not investigate whether CDPK1 knockdown affected its phosphorylation. Instead, we examined whether knockdown of *mtip* or *myoa* by promoter swap would be sufficient to generate the phenotype of the of P_*clag*_-*cdpk1-gfp* mutant. This time 1.5 kb of upstream sequence from *ama1* (Figure S2), an invasion-related gene transcribed in mid-schizonts that is silent in gametocytes and ookinetes, were used to achieve a stage specific gene knockdown in ookinetes (Siden-Kiamos et al., 2011). P_*ama1*_-*myoa* and P_*ama1*_-*mtip* parasite clones developed normally during asexual blood stages but produced ookinetes that lacked detectable amounts of the respective proteins (Figure 3A, lanes 3 and 4). Interestingly, in P_*ama1*_-*mtip* ookinetes, MyoA was also fully depleted, indicating that MyoA is stabilized by its light chain. The reverse was not true, however, since MTIP persisted in the P_*ama1*_-*myoa* mutant. Importantly, neither P_*ama1*_-*myoa* nor P_*ama1*_-*mtip* ookinetes had the morphological defects seen in the CDPK1 knockdown lines (Figure 3B), and both were able to bend and twitch, although neither could glide (Figure 3C and Movie S1). These data confirm the suspected functions of MyoA and MTIP in gliding motility of *Plasmodium* but clearly establish that a functional motor is not required for ookinete differentiation. Depletion of MTIP and MyoA cannot, therefore, account for the developmental arrest of P_*clag*_-*cdpk1-gfp* ookinetes.

### GAP45 Has a Unique Essential Function Early in Ookinete Formation

GAP45 is another member of the motor complex and a substrate for CDPK1 in vitro. We were unable to assess GAP45 protein levels in P_*clag*_-*cdpk1-gfp* ookinetes by western blot, because available antisera against *P. falciparum* GAP45 do not cross-react with *P. berghei*. To investigate whether CDPK1 controls ookinete development through GAP45, we therefore downregulated *gap45* in sexual stages by promoter swap (Figure S2C, S2G, and S2I). Gametogenesis and fertilization in the P_*ama1*_-*gap45* mutant were unaffected, but zygotes remained spherical and failed entirely to initiate transformation into ookinetes as judged by light microscopy (Figure 3B). To confirm that this phenotype was indeed due to the loss of GAP45, we complemented the mutant by additionally inserting a cassette expressing *P. falciparum* GAP45 from the *P. berghei gap45* promoter into the P_*ama1*_-*gap45* promoter swap construct (Figures S2D and S2H). Genomic integration of this allele at the *gap45* locus led to the expression of Pf*gap45* mRNA and protein in schizonts and ookinetes (Figures 3D and S2I). Complementation restored the formation of ookinetes (Figure 3E), which were fully motile (data not shown), confirming that the unexpected block in zygote development is a true loss-of-function phenotype of *gap45*. We therefore examined the ultrastructure of P_*ama1*_-*gap45* zygotes 20 hr after fertilization and observed an intact apical complex featuring an apical collar, inner membrane complex, microtubules, and micronemes (Figure 3F). However, these structures were detached from the plasmalemma and were “free floating” in the cytoplasm of a round zygote. These data are consistent with the recently identified role for GAP45 in *T. gondii* to provide a structural link between the IMC and the plasma membrane (Frénal et al., 2010). Unlike other components of the motor complex, GAP45 does thus appear to play a role in ookinete formation, but the developmental role of CDPK1 cannot be explained by GAP45 alone, as the phenotype of the arrested ookinetes is very different in the two knockdown lines.

### Disrupted Expression of Important Ookinete Proteins in P_*clag*_-*cdpk1-gfp* Parasites

Since functions of the motor complex provided no obvious mechanism for the transformation defect of the P_*clag*_-*cdpk1-gfp* mutant, we turned to quantitative mass spectrometry to ask whether the expression of additional proteins was altered. Using at first a conventional experimental design for stable isotope labeling in culture (SILAC) (Ong et al., 2002), we pulse-labeled wild-type ookinetes with heavy amino acids (^13^C-_6_,^15^N_2_ L-lysine and ^13^C_6_,^15^N_4_ L-arginine) for 16 hr after gametocyte activation to identify proteins synthesized de novo during this period. Samples from seven biological replicates were separated on protein gels and each replicate gel lane was divided into fractions, each of which was analyzed by LC-MS/MS. In this experiment, 1,424 parasite proteins were identified by MS, representing a 1.86-fold increase in ookinete proteome coverage by MS compared to published data sets (Hall et al., 2005). Stable isotope incorporation could be measured for 1,336 of these proteins (Table S1, part A). The labeled proteins fell into two surprisingly distinct classes (Figure S3B). One thousand, one hundred sixty-two proteins had an incorporation rate below 20%, which we hypothesize are housekeeping proteins that largely pre-exist in the macrogametocyte and turn over slowly in the differentiating ookinete. A much smaller number of 91 proteins showed incorporation rates >80%, suggesting that these proteins were actively synthesized only after fertilization. The latter group included most proteins known to be involved in motility and invasion and showed a 6.8-fold enrichment of genes, whose messages are translationally repressed in gametocytes, as described by Mair et al. (2010).

**Figure 4:**
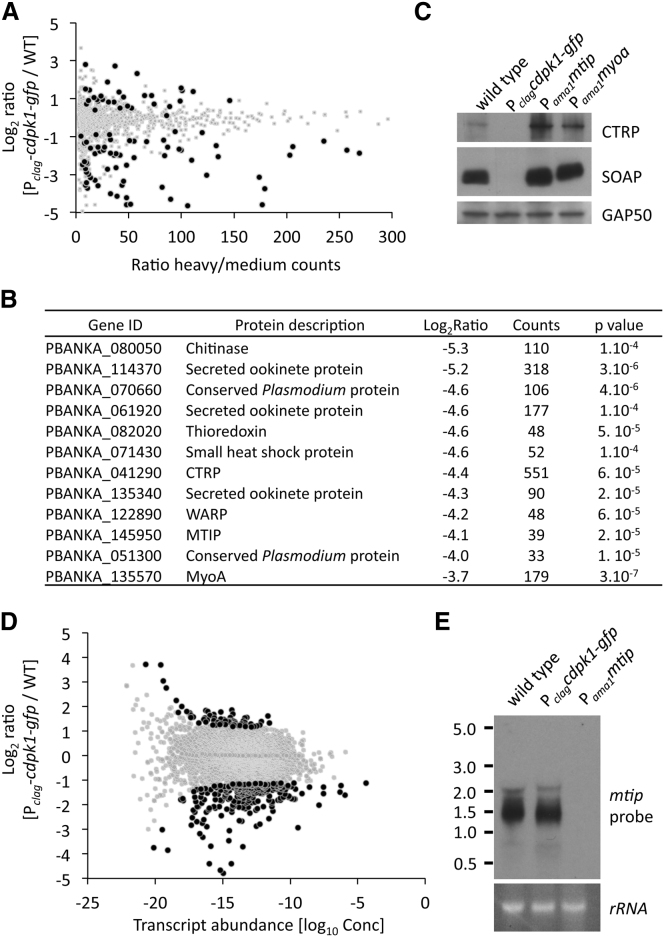
Global Analysis of Transcripts and Proteins in P_*clag*_-*cdpk1-gfp* Ookinetes (A) Normalized protein ratios are plotted against the heavy/medium ratio counts for each protein. The spread of the cloud is lower at high abundance, indicating that quantification is more precise. The data points are colored by significance across five biological replicates: black, p < 0.001; gray, p > 0.001. (B) Proteins depleted in P_*clag*_-*cdpk1*-*gfp* mutant are mainly associated with ookinete specific functions. Listed are the top twelve annotated proteins depleted in the mutant. (C) Western blot analysis of SOAP and CTRP protein in lysates of ookinetes. (D) Normalized transcript ratios are plotted against transcript abundance (log_10_Conc). Colors show significance: black, p < 0.001; gray, p > 0.001. (E) Northern blot showing equal transcript levels for *mtip* in WT compared to P_*clag*_-*cdpk1-gfp* ookinetes. Control lane: P_*ama1*_-*mtip* ookinetes, which lack *mtip* mRNA. See also Figure S3 and Table S1.

Recognizing that in a conventional SILAC experiment incomplete isotope incorporation would prevent protein quantification, we used a triple isotope labeling strategy (Blagoev et al., 2004) to study the P_*clag*_-*cdpk1-gfp* mutant (Figure S3A). Wild-type ookinetes were grown in the presence of “medium” (D_4_ L-lysine and ^13^C_6_ L-arginine) amino acids, and mutant ookinetes were grown with “heavy” (^13^C-_6_,^15^N_2_ L-lysine and ^13^C_6_,^15^N_4_ L-arginine) spectra were acquired and 2,161 proteins were identified at a 1% false discovery rate. Four hundred sixty-five were mouse proteins and 1,696 were *P. berghei* proteins, of which protein ratios could be calculated for 1,498 (88%). Rigorous filtering was performed to provide robust quantification for 967 proteins, 70 of which (7.2%) were significantly less represented among the labeled proteins in the mutant and 34 of which (3.5%) were more represented (p < 0.01; Figure 4A and Table S1, part B). MyoA and MTIP were among the most severely affected proteins (Figure 4B), while GAP45 and GAP50 were not significantly reduced (Table S1, part B). Other proteins depleted in P_*clag*_-*cdpk1-gfp* ookinetes included components of the subpellicular network, such as alveolins, microneme proteins, circumsporozoite- and TRAP-related protein (CTRP), and secreted ookinete adhesive protein (SOAP). Where antibodies were available, we confirmed the absence of these proteins by western blot (Figure 4C). The shorter list of upregulated proteins in P_*clag*_-*cdpk1-gfp* ookinetes was not obviously enriched for a functional category of genes but, interestingly, it contained both *Plasmodium* homologs of the HoBo (Homolog of Bruno) family of mRNA binding proteins (PBANKA_113570 and PBANKA_103270), both of which also form part of the DOZI protein complex (Mair et al., 2010).

### Dysregulation of Proteins Does Not Correlate with Transcript Abundance

**Figure 5:**
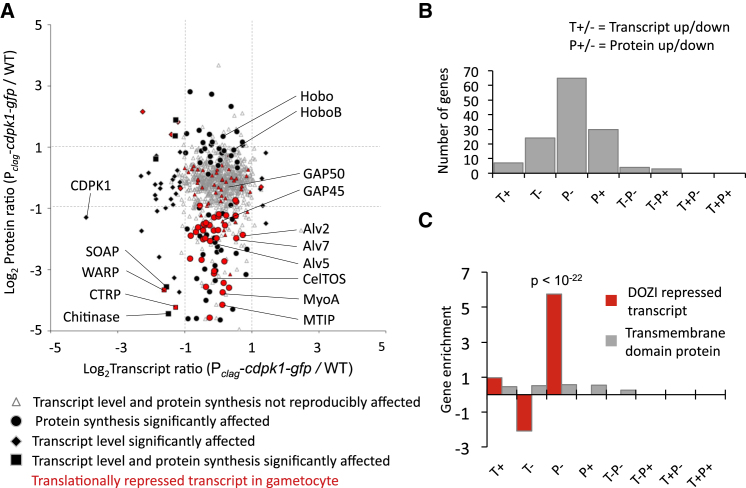
P_*clag*_-*cdpk1-gfp* Ookinetes Are Depleted of Posttranlationally Regulated Proteins (A) The degree of deregulation of transcript (T) and protein (P) in P_*clag*_-*cdpk1-gfp* ookinetes are plotted against each other for all quantified gene products. Significantly deregulated proteins or transcript (p < 0.001) are shown with black symbols. Not all large effects reach statistical significance. Genes with independent evidence for translational repression are shown in red. (B) Numbers of significantly dysregulated genes in P_*clag*_-*cdpk1-gfp* ookinetes by category: P–, protein down, transcript not affected; T–P+, transcript down, protein up; etc. (C) Enrichment by category of dysregulation is shown for known DOZI repressed transcripts. Proteins with predicted transmembrane domains served as a control. See also Table S2.

To assess whether the lack of a small subset of key proteins from P_*clag*_-*cdpk1-gfp* ookinetes was a consequence of changed transcription, we compared the transcriptomes of wild-type and mutant ookinetes by RNA sequencing. Two hundred fifty-two of 4,692 transcripts (5.5%) were significantly less abundant in the mutant, while 65 (1.3%) were more abundant (p < 0.001; Figure 4D and Table S1, part C). Despite the depletion of MyoA and MTIP protein from the P_*clag*_-*cdpk1-gfp* ookinetes, their mRNA levels were unchanged, an observation we confirmed for *mtip* mRNA by northern blot analysis (Figure 4E). Transcriptional changes do not therefore appear to underlie the dysregulation of protein levels. To assess this across the two global data sets, we plotted the degree of dysregulation of transcript and protein against each other (Figure 5A) and assigned all dysregulated genes to categories according to the direction and degree of their alteration (Figure 5B). Only five genes showed evidence for significant coregulation of transcript and protein between wild-type and mutant (listed in Table S2). The single largest dysregulation category contained 65 genes, all of which had reduced protein levels despite normal mRNA counts (Figure 5B and Table S2). This group was characterized by an 8-fold enrichment in genes whose mRNAs were shown previously to rely on either the DOZI helicase or CITH to be stabilized in gametocytes (Mair et al., 2006, 2010). It is thought that these transcripts associate with mRNP complexes defined by DOZI and CITH to achieve stability and prevent early translation. The enrichment for suspected translationally repressed transcripts among the dysregulated proteins of the P_*clag*_-*cdpk1-gfp* was highly significant (p < 10^−22^). However, some known translationally controlled proteins were not dysregulated, including the major ookinete surface protein p28, which was expressed to similar levels in wild-type and mutant as confirmed by western blot analysis (Figure S1). The data therefore suggest that CDPK1 is required for the translational activation of only a subset of repressed mRNAs in the ookinete. However, recognizing that MyoA was depleted from mutants lacking MTIP (Figure 3A), we also had to consider the alternative hypothesis that components of the motor complex are depleted as a result of enhanced turnover following their destabilization in the absence of CDPK1.

### CDPK1 Is Required for Translation of MTIP mRNA

**Figure 6:**
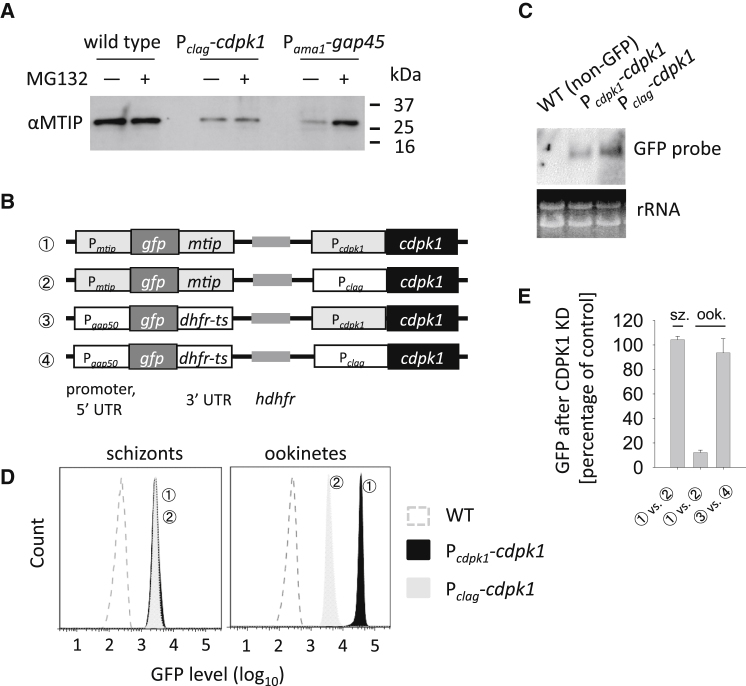
Translational Derepression of a GFP Reporter Controlled by *cdpk1* (A) Western blot comparing the effect of proteasome inhibition on stabilization of MTIP after knockdown of GAP45 or CDPK1. (B) Schematic of four reporter constructs introduced into the *cdpk1* locus by double homologous recombination. (C) Northern blot showing *gfp* transcript levels are not reduced by knockdown of *cdpk1*. (D) Impact of *cpdk1* knockdown on translational derepression of GFP. Average GFP levels as determined by flow cytometry. (E) Representative fluorescence levels as determined by flow cytometry for P_*mtip*_*-gfp* parasites, comparing P_*cdpk1*_*-cdpk1* and P_*clag*_*-cdpk1* in schizonts and ookinetes. Error bars represent ± SD; n = 3.

To distinguish between these possibilities, we first examined whether in the absence of CDPK1, MTIP is destabilized and degraded by the proteasome. In the GAP45 knockdown line, the proteasome inhibitor MG132 restored MTIP to wild-type levels (Figure 6A), showing that the half-life of MTIP is reduced outside of the motor complex. In marked contrast, in the CDPK1 knockdown line, MG132 failed to restore MTIP to the ookinete, suggesting reduced synthesis, not enhanced turnover, as the primary reason for MTIP depletion from this mutant. Since knockdown of CDPK1 had no impact on *mtip* mRNA levels, we conclude that CDPK1 is required for the regulated derepression of the *mtip* mRNA.

### CDPK1 Regulates Derepression of mRNA via Their 3′ or 5′ UTRs

The sequence motifs regulating translation repression by the DOZI protein complex reside within the untranslated regions of posttranscriptionally regulated mRNAs (Braks et al., 2008). We therefore predicted that, if CDPK1 directly regulates derepression of mRNA, the 5′ and 3′ untranslated regions (UTRs) of *mtip* mRNA should be necessary and sufficient to place the translation of an unrelated reporter protein under the control of CDPK1. To test this idea reporter constructs were generated, in which GFP was placed under the control of 5′ and 3′ flanking sequences derived either from *mtip*, or from *gap50*, a control gene we found to be translated independently of CDPK1 (Figures 2A and 5A). These reporter cassettes were inserted upstream of the *cdpk1* gene as part of a construct that either left the *cdpk1* promoter intact or exchanged it for P_*clag*_, in order to either maintain or downregulate CDPK1 expression (Figure 6B). When GFP was flanked by UTRs from MTIP, knockdown of CDPK1 had no impact on *gfp* mRNA levels (Figure 6C), but strongly reduced GFP protein levels in ookinetes, as assessed by flow cytometry, (Figures 6D and 6E). By contrast, when GFP was flanked by UTRs from a nonrepressed gene (*gap50*), knockdown of CDPK1 had no impact on GFP protein levels (Figure 6E). In schizont stages, where MTIP is not known to be translationally regulated and where the CDPK1 promoter exchange does not have a phenotype, GFP fluorescence was independent of the promoter driving CDPK1 expression (Figure 6D). These data therefore provide evidence for a direct link between the CDPK1 protein kinase and the derepression of *mtip* mRNA that is controlled by sequences flanking the *mtip* open reading frame.

## Discussion

Suggestions that CDPK1 may be an important regulator of the *Plasmodium* motor complex have been based primarily on its ability to phosphorylate MTIP and GAP45 in vitro and on its association with the plasmalemma in mature *P. falciparum* schizonts (Green et al., 2008; Kato et al., 2008). The idea was further supported by a cluster analysis of gene expression profiles across *Plasmodium* species and stages showing that *cdpk1* is coregulated with motility and invasion-related genes (Kato et al., 2008). A functional analysis of *cdpk1* by reverse genetics has, however, been prevented by its essential role in blood stages. In the current study we have identified functions of CDPK1 by investigating the sexual and early mosquito stages of the murine malaria parasite *P. berghei*.

A CDPK1-GFP fusion protein that we expressed from the *cdpk1* promoter within the *cdpk1* chromosomal locus was present at all life-cycle stages, including the gametocyte, the zygote, and the early oocyst, neither of which possess a functional motor complex. These expression data therefore hinted at functions for CDPK1 in noninvasive stages, which we confirmed by knocking down *cdpk1* expression in a sexual stage specific manner. A delay in the emergence of microgametocytes, that otherwise exflagellated normally, suggested a function of CDPK1 specifically in host cell lysis. Whether emergence of macrogametocytes was also delayed, could not be assessed reliably. Activation of *P. berghei* gametocytes by xanthurenic acid triggers within 10 s the release of Ca^2+^ from intracellular stores, which is a requirement for all subsequent events of gamete formation, including parasite emergence from the host erythrocyte (Billker et al., 2004). CDPK4 is the Ca^2+^ effector kinase critical for the initiation of DNA replication during the first minute after microgametocyte activation, but this kinase was not required for the emergence of gametocytes from their host cells, leading to the suggestion that other calcium effectors were involved (Billker et al., 2004). Emergence relies on the secretion of osmiophilic bodies from the gametocyte cytosol, which may contain enzymatic mediators of host cell lysis, and it is tempting to speculate that CDPK1 may regulate this process. In contrast to a complete knockout of a secreted host cell lytic factor (Talman et al., 2011), emergence of the stage-specific *cdpk1* knockdown was merely delayed, but not completely prevented, suggesting that either additional signaling pathways can regulate emergence, or that any residual CDPK1 activity in the mutant was sufficient to eventually trigger emergence.

The delay in microgamete emergence did not affect the rate at which macrogametocytes of the *cdpk1* knockdown mutant were fertilized and ookinete formation was initiated. We therefore concluded that the developmental block of the P_*clag*_-*cdpk1* mutant during ookinete formation was due to a second function of CDPK1 unrelated to its role in microgametocyte emergence, and that this block was most likely responsible for the nearly complete inability of the mutant to infect mosquitoes. The loss of MyoA and MTIP proteins from P_*clag*_-*cdpk1* parasites confirmed a link between CDPK1 and the motor complex, but also prevented us from studying phosphorylation of MTIP in vivo. Furthermore, since GAP45 in the mutant was no longer part of a heterotrimeric motor complex, we reasoned that no useful insights would be gained from analyzing its phosphorylation status. Instead, we investigated whether MTIP and MyoA are required for zygote transformation, in which case their loss could provide a mechanistic explanation for the differentiation defect of P_*clag*_-*cdpk1* zygotes. Through the knockdown of MTIP, we provide genetic evidence that the myosin light chain is essential for ookinete gliding, but not for development. We additionally confirm recent data by Siden-Kiamos et al. (2011) showing the same for MyoA itself.

GAP45, in contrast, plays an important role in ookinete morphogenesis. The complete lack of cohesion between plasmalemma and IMC in the stage-specific knockdown mutant demonstrates that GAP45 is a critical tethering factor between these membranes and provides a striking confirmation of recent findings from a tetracycline-induced knockdown in *T. gondii* tachyzoites (Frénal et al., 2010). In the P_*clag*_-*cdpk1* mutant GAP45 is expressed, and transmission electron microscopy revealed no defect in the spacing between the plasmalemma and the IMC of the developmentally arrested ookinetes. We therefore conclude that GAP45 does not rely on MTIP, MyoA or CDPK1 to reach its location in the supra-alveolar space, or to become palmitoylated and insert into the plasmalemma and IMC membrane. In *T. gondii* tachyzoites, MyoA, MTIP, and GAP45 assemble into a heterotrimeric complex prior to their association with GAP50 in the IMC (Gilk et al., 2009). It is clear, however, that in ookinetes GAP45 can fulfill its structural role outside of this complex, which may serve primarily to deliver and anchor MyoA and MTIP to their location in the supra-alveolar space.

For a deeper understanding of its molecular phenotype we conducted quantitative proteome and transcriptome analyses of the P_*clag*_-*cdpk1* mutant. The largest class of 65 dysregulated genes was characterized by a reduced protein level despite normal amounts of transcript, and was also highly enriched for genes whose mRNAs are known to be stabilized and translationally repressed in DOZI- or CITH-containing mRNP complexes in zygotes. These data suggested CDPK1 may be required to lift the translational block on these mRNAs. A further four genes (*ctrp*, *soap*, *warp*, and *cht*) were reduced at both transcript and protein levels. All four encode important microneme proteins shown previously to be regulated by AP2-O (Yuda et al., 2009), a transcriptional regulator of the ApiAP-2 family of DNA binding proteins. Interestingly, *ap2-o* mRNA is itself DOZI dependent (Mair et al., 2006), raising the possibility that transcriptional downregulation of major microneme genes in the P_*clag*_-*cdpk1* mutant may be an indirect consequence of translational dysregulation of AP2-O. We were unable to measure with confidence AP2-O levels to test this hypothesis. In addition to microneme proteins and elements of the motor complex, structural components of the inner membrane complex, such as alveolins, as well as proteins of unknown function, were reduced in the P_*clag*_-*cdpk1* mutant, which collectively might explain the developmental block in zygote development.

In the presence of largely unchanged transcript abundances, the reduced levels of many ookinete proteins upon CDPK1 knockdown could be due to reduced translation or increased protein turnover. The latter would be highly plausible for proteins of the motor complex, since we observed that MyoA was lost upon downregulation of MTIP, and that MTIP was much reduced after knockdown of GAP45. In the latter case, addition of a proteasome inhibitor stabilized MTIP, suggesting that enhanced turnover in the absence of a stabilizing protein complex is probably responsible for its loss from the mutant. In marked contrast, proteasome inhibition failed to restore MTIP in CDPK1 knockdown ookinetes, indicating that CDPK1 controlled MTIP at the level of translational activation. Translational repression in *P. berghei* macrogametocytes can be mediated by a uridine-rich 47-mer in the 3′UTR of silenced transcripts, which is sufficient to transfer repression to a GFP reporter protein (Braks et al., 2008). Using a similar reporter construct as in the previous study, we have shown here that 3′ and 5′ UTRs from MTIP can bring translational activation of GFP in the ookinete under the control of CDPK1. This presents striking evidence for a link between CDPK1 and translational activation of repressed transcripts.

Translational repression in female gametocytes is mediated by *cis*- and *trans*-acting factors that are closely related to those used by female germline cells in metazoans (Braks et al., 2008; Mair et al., 2010). How silenced *Plasmodium* mRNAs are activated for translation is not understood, but the process may well involve phosphorylation. In *Xenopus* oocytes, for instance, activation of some mRNAs involves phosphorylation of the RNA binding protein CPEB (cytoplasmic polyadenylation element binding) by mitotic kinase aurora A (Stitzel and Seydoux, 2007). Although translational repression in *Plasmodium* is clearly an ancient mechanism of post-transcriptional gene control, not all proteins involved are conserved and the *Plasmodium* genome encodes no obvious orthologs for CPEB or aurora A. The increased abundance of both Bruno-like putative mRNA binding proteins upon CDPK1 knockdown is intriguing, as is the significant depletion of a putative elongation initiation factor (PBANKA_052310; p = 2.2 × 10^−5^). However, further work is required to establish whether CDPK1 regulates translational activation directly, for instance by phosphorylating a component of the silencing complex.

Notwithstanding its putative role in regulating the motor complex, our data demonstrate that CDPK1 has multiple key functions during early sexual development and mosquito transmission of *P. berghei*, linking fertilization to the regulated translational activation of a subset of mRNAs in the developing ookinete. Future work will have to investigate the nature of the link by identifying CDPK1 substrates and interacting proteins. Our data do not rule out additional roles for CDPK1 in regulating the motor or its assembly directly. They rather raise the intriguing possibility that the same CDPK1 dependent pathways control ookinete development and gliding at the level of translation and through posttranslational modification.

## Experimental Procedures

### Parasite Maintenance and Transmission

All animal experiments were conducted under a license from the UK Home Office in accordance with national and European animal welfare guidelines. *P. berghei* ANKA parasites were maintained in female Theiler's Original outbred mice and *A. stephensi* (strain SD500) mosquitoes. Infected mosquitoes were maintained at 19°C on a solution of fructose and oocyst numbers were counted on dissected midguts on day 10, sporozoite numbers in salivary glands were determined on day 21 postinfection.

### Transfection Vectors and Protocols

A detailed description of how transfection vectors were constructed be found in the Supplemental Experimental Procedures. Oligonucleotides used are shown in Table S3. Schizonts for transfection were purified from overnight cultures and transfected with 5 μg linearized DNA as described (Janse et al., 2006). Electroporated merozoites were allowed to reinvade reticulocytes at 37°C for 20 min before being injected intraperitoneally into a naive mouse. Resistant parasites were selected by pyrimethamine supplied in the drinking water at 70 mg/liter or WR99210 administered intraperitoneally daily for 4 days at 16 mg/kg body weight.

### Phenotype Analysis

Exflagellation of male gametocytes was monitored by adding 10 μl tail blood from a high-gametocytaemia mouse into 10 μl ookinete medium (RPMI1640 containing 25 mM HEPES, 20% FCS, 100 μM xanthurenic acid [pH 7.4]) and observing exflagellation centers under a light microscope with a 40× objective. For quantification, the ratio of unemerged activated male gametocytes (with intracellularly beating flagella) to emerged male gametocytes (with extracellular male gametes) was determined in four 2 min intervals starting at 12 min postactivation. For ookinete cultures 1 volume of heparinized mouse blood was inoculated into 9 volumes of ookinete medium and incubated for 20 hr at 19°C. The conversion efficiency of female gametocytes to mature ookinetes was determined in live cultures by adding anti-p28-Cy3 monoclonal antibody and calculating the ratio of mature, p28-positive ookinetes compared to all other p28-positive, round cells (unfertilized females and developmentally arrested zygotes) from an ookinete culture. Ookinete motility was observed as described previously (Moon et al., 2009) in a mixture of ookinete medium and Matrigel (BD Biosciences). High-magnification time-lapse videos of single ookinetes were acquired on a Leica M205A at 19°C. Movies were analyzed with Fiji and the Manual Tracking plugin (http://fiji.sc/wiki/index.php/Manual_Tracking). For biochemical analysis, ookinetes were purified from overnight cultures using paramagnetic anti-mouse IgG beads (Dynabeads, Invitrogen) coated with anti-p28 mouse monoclonal antibody (clone 13.1). Transmission electron microscopy of ookinetes was performed as described previously (Moon et al., 2009).

### SILAC Labeling and Quantitative Mass Spectrometry Analysis

Ookinetes were cultured in customized RPMI1640 medium lacking arginine and lysine (Invitrogen) in the presence of ^13^C-_6_,^15^N_2_ L-lysine and ^13^C_6_,^15^N_4_ L-arginine or D_4_ L-lysine and ^13^C_6_ L-arginine for 16 hr from the moment of gametocyte activation. Ookinetes were then purified and proteins were extracted as previously described (Emes et al., 2008). Forty micrograms of total protein was used for each replicate. Protein gels were stained overnight with colloidal Coomassie blue (Sigma). Each lane was excised and cut into 24 bands that were destained and in-gel digested overnight using trypsin (sequencing grade; Roche). Peptides were extracted from gel bands twice with 50% acetonitrile/0.5% formic acid and dried in a vacuum centrifuge. Peptides resuspended in 0.5% formic acid were analyzed online with an Ultimate 3000 Nano/Capillary LC System (Dionex) coupled to an LTQ Orbitrap Velos hybrid mass spectrometer (Thermo Electron) equipped with a nanospray ion source. See the Supplemental Experimental Procedures for details of data acquisition and analysis.

For identification of significant differences in protein expression, results from a MaxQuant analysis were further analyzed by Perseus (version 1.2.0.17). Proteins with a minimum ratio count of three per group were taken forward for analysis and protein ratios were normalized to the median ratio for each sample and log2 transformed. t tests were performed with Benjamini-Hochberg correction for multiple hypothesis testing to achieve a 1% false discovery rate for identification of significantly altered proteins in the P_*clag*_-*cdpk1-gfp* mutant. Incorporation of labeled amino acids and relative protein levels between the WT and *cdpk1* mutant were determined in seven and five independent biological replicates, respectively.

Enrichment or depletion of proteins in regulation categories was calculated according to the hypergeometric distribution: cumulative hypergeometric probabilities were calculated for enrichment and depletion for each group of proteins (DOZI+, DOZI–, TM) and each regulation category (T+, T–, P+, P–, T–P–, T–P+ and T0P0). Protein groups were labeled as “enriched” if the probability of finding the observed number of occurrences or more in a given regulation category was < 0.05. The probability for finding a protein of any given category in a sample was based on the numbers of detected proteins in total and in each respective category.

### RNA Sequencing

Approximately 10 **μ**g total RNA were extracted from purified ookinetes for each replicate using the RNeasy kit (QIAGEN). Depletion of ribosomal RNAs and highly abundant transcripts, and sequencing library construction were performed as previously described (Otto et al., 2010). The library was end-sequenced (54 nt) on an Illumina GAII instrument. Paired-end reads were mapped to the *P. berghei* ANKA genome obtained from GeneDB with the Tophat alignment tool (Trapnell et al., 2009). Properly mapped reads were counted for each annotated gene region (plus 200 bp up-/downstream margin) using custom Perl scripts to obtain a matrix of gene expression levels, which was subjected to differential expression analyses using Bioconductor (v.2.6) packages EdgeR (Robinson et al., 2010) and DESeq (Anders and Huber, 2010). In EdgeR, raw read counts below 5 were discarded and remaining counts normalized using the “weighted trimmed mean of M values” (TMM) method. Fisher's exact test with common dispersion correction and the Benjamini & Hochberg FDR method were used for calling differentially expressed genes. Default methods for normalization and differential expression identification were used with the DESeq package.

### Flow Cytometry Analysis

Purified schizonts and ookinetes were stained for 15 min with Hoechst 33342 DNA dye. Samples were examined with a 488 nm laser and a 355 nm laser on a BD LSR Fortessa flow cytometer. BD FACSDiva software was used to collect 10,000 events for each sample. The data collected was further analyzed with FlowJo (Tree Star, Ashland, Oregon). The schizont and ookinete populations were identified by Hoechst stain, and average GFP fluorescence was then determined in the gated schizont or ookinete population.

## Supplemental Information

Table S1. Data Tables from Proteome and Transcriptome Analyses, Related to Figure 4

Table S2. Gene Products in P_*clag*_-*cdpk1-gfp* Ookinetes by Dysregulation Category, Related to Figure 5

Table S3. Oligonucleotides Used in This Study, Related to Figure 1

Movie S1. Gliding Motility in a Panel of Mutant Ookinetes, Related to Figure 3

Document S1. Supplemental Experimental Procedures and Figures S1–S3
