# Supplementary material for: A Plasmodium Calcium-Dependent Protein Kinase Controls Zygote Development and Transmission by Translationally Activating Repressed mRNAs
Source: Cell Host Microbe. 2012 Jul 19;12(1-10):9–19. doi: 10.1016/j.chom.2012.05.014 (PMC3414820; doi:10.1016/j.chom.2012.05.014)
Supplement: Table S3. Oligonucleotides Used in This Study, Related to Figure 1 [file mmc3.pdf]

Table S3. Oligonucleotides Used in This Study, Related to Figure 1

PCR product	Forward primer		Reverse primer	
	**Name**	**Sequence**	**Name**	**sequence**
CDPK1 for GFP tagging	olOB500	atatggtaccgaaggtggagaattatttgagc	olOB501	atatgggcccaaatgttttatggtcacaaattttgtg
clag promoter	olSS822	acacggtaccctgatatttatgagtattccatg	olSS823	agagctcgagtatattacgaattgacaccatttttc
ama1 promoter	olSS1009	atatggtacccccacatacatggatataca	olSS1010	agagctcgagttttatatcgttttattttattaatatttt
5'HR CDPK1	olSS820	acacccgcggttactgaatactcgtatgtgtta	olSS821	acacctgcagacactcatcaaatttatgcctatt
3'HR CDPK1	olSS824	agagctcgagaatggggtgtaatcaaagtaaaagtg	olSS840	agcggccgcgatatcaaatttatgcctattaataatttgc
5'HR MyoA	olSS1066	gagaccgcggcattgaagaattgtattttgttac	olSS1067	gagactgcagcgagaaacgagaaaaatcaaaatt
3'HR MyoA	olSS1068	gagactcgagatggctgttacaaatgaggaat	olSS1069	gagagcggccgcgttaacgccatgtaaatttgataaag
5'HR MTIP	olSS1016	gagaccgcggttactatatatgtgtagaaatg	olSS1017	gagactgcagttttgcgatatatatttttttaaattaa
3'HR MTIP	olSS1018	gagactcgagatggaacaacaatgccatat	olSS1019	gagagcggccgcgatatcctcgcaaaataatttataat
5'HR GAP45	olSS927	gagaccgcggcaggaatatcttatatagc	olSS928	gagactgcaggcaaatcggtataatgtctta
3'HR GAP45	olSS929	gagactcgagatgggaagcagatgttcaa	olSS930	gagagcggccgcgatatcggataaatcaatctttc
CDPK1 gene for complementation	olSS880	atatccgcggacatatgcacaacttccgtttttttatg	olSS883	acacgctagcttttggcttccttcaaattttc
RT-PCR for PbGAP45	olSS1176	tgaaggtgatcaaaatg	olSS1177	aattccagtagcatcg
RT-PCR for PfGAP45	olSS1178	gaactggatgaacagaa	olSS1179	ggcaccagtggcttca
RT-PCR for PbGAP50	olSS1054	taccattattaaaagaagc	olSS1055	ataaaaaagaggatacac
gap50 5'utr	olSS1083	agagctcgagaattattgtctaaaatcaattc	olSS1084	agagctcgaggttcactaaataaatttaaa
mtip 5'utr	olSS1113	gagaccgcggattatatttagcttttttctt	olSS1114	ctcgagttttgcgatatatatttttttaaattaa
mtip 3'utr	olSS1125	ctcgagattttataatctatattttttacaat	olSS1126	catgtaacagataaacttaa
GFP cds	olSS1118	ctcgagatggtgagcaagggcga	olSS1120	ctcgagttacttgtacagctcgtc
Genotyping	olSS1020	attatatttagcttttttctt		
Genotyping	olSS1021	gattataaattattttgcgag		
Genotyping	olSS1009	atatggtacccccacatacatggatataca		
Genotyping	olSS858	tctactttatttgctaattctgg		
Genotyping	olSS1070	atacatacttgcatgtgtgt		
Genotyping	olSS1071	ggttagctgccataattgct		
Genotyping	olSS935	gtctatatgcaatgaagtgc		
Genotyping	olSS936	gtgtgtgggtaatgaaaacaa		
Genotyping	olSS1179	ggcaccagtggcttca		

HR = homology region
