# Supplementary material for: A Plasmodium Calcium-Dependent Protein Kinase Controls Zygote Development and Transmission by Translationally Activating Repressed mRNAs
Source: Cell Host Microbe. 2012 Jul 19;12(1-10):9–19. doi: 10.1016/j.chom.2012.05.014 (PMC3414820; doi:10.1016/j.chom.2012.05.014)
Supplement: Document S1. Supplemental Experimental Procedures and Figures S1–S3 [file mmc5.pdf]

Cell Host & Microbe, ***Volume 12***

Supplemental Information

A *Plasmodium* Calcium-Dependent Protein Kinase Controls Zygote Development and Transmission by Translationally Activating Repressed mRNAs

Sarah Sebastian, Mathieu Brochet, Mark O. Collins, Frank Schwach, Matthew L. Jones, David Goulding, Julian C. Rayner, Jyoti S. Choudhary, and Oliver Billker

Supplemental Experimental Procedures

Genetic modification vectors

For c-terminal GFP tagging of the the *cdpk1* genomic locus, we first amplified by PCR the terminal 1.5 kb of *cdpk1* without the stop-codon, using oligonucleotides ol500 and ol501 (Table S3). The fragment was cloned in frame into the ApaI/KpnI sites of the EGFP-tagging vector p277 . For transfection, this construct (pSS312) was linearised at a natural HpaI site within the *cdpk1* sequence. For the double-crossover promoter-exchange plasmids, we first constructed parent vectors P_{clag} and P_{ama1} containing the respective promoters. For P_{clag}, 1.8 kb of upstream sequence from the *clag* gene (PBANKA_083630) was cloned downstream of the *hdhfr* cassette in pDEFhDHPEA (kindly provided by Blandine Franke-Fayard). For P_{ama1}, 1.5 kb of the upstream sequence of the *ama1* gene (PBANKA_083630) were similarly cloned. The choice of promoter proved crucial for the success of knock down experiments. The promoter of the *clag* gene was a suitable replacement for the endogenous CDPK1 promoter, resulting in sufficient (albeit much decreased) CDPK1 levels in schizonts, and in a pronounced knock-down of kinase expression in the early mosquito stages. Notably to down regulate the motor complex components MTIP, MyoA, and GAP45 in ookinetes, we resorted to the use of the *ama1* promoter, since a *clag* promoter exchange did not result in viable blood stage parasites after transfection. These observations highlight the importance of maintaining appropriate protein levels under the new promoter in the asexual parasite stages, where the gene of interest has an essential function. The P_{clag}-*cdpk1* vector pSS367 was made by inserting a *cdpk1* 5' homology region upstream of the *hdhfr* cassette in P_{clag} (consisting of 500 bp of *cdpk1* upstream sequence), and a 3' homology region downstream of the clag promoter (consisting of the first 500 bp of *cdpk1* coding sequence). Equivalent construct design was used for P_{ama1}-*myoA* (pSS368), P_{ama1}-*mtip* (pSS369), and P_{ama1}-*gap45* (pSS370).

For the *cdpk1* complementation construct (pSS371), we used a parent vector for single-crossover insertion into the *c/d-ssu* locus as described previously (van Spaendonk et al., 2001). The complete *cdpk1* gene including 2 kb of upstream promoter sequence, but omitting the stop codon, was cloned upstream in frame with an enhanced GFP coding sequence, that was followed by a *dhfr/ts* 3'UTR. After insertion into the *c/d-ssu* locus, this vector expresses GFP-tagged CDPK1 from the *cdpk1* promoter. To complement with *P. falciparum gap45,* we started with the vector for P_{ama1}-*gap45* promoter exchange (pSS370) and inserted the *Pfgap45* coding sequence plus the *Pbdhfr/ts* 3'UTR downstream of the 5' homology region containing the *Pbgap45* promoter that served as 5' homology region for recombination. As a result of insertion by double crossover, PfGAP45 is expressed from the *P. berghei gap45* promoter, and PbGAP45 from the *ama1* promoter.

To make reporter constructs expressing GFP under the control of either *gap50* or *mtip* regulatory sequences, we first constructed reporter cassettes in the subcloning plasmid

pGEM (Promega). For *gap50*-controlled GFP, we cloned the GFP coding sequence plus the *dhfr 3'* UTR downstream of the *gap50* promoter (comprising 1 kb of *gap50* upstream sequence). For *mtip*-controlled GFP expression, we cloned the GFP coding sequence between 5' and 3' UTRs from the *mtip* gene, comprising 780bp and 760bp, respectively. The completed reporter cassettes were then amplified by PCR from their subcloning plasmids and inserted into two different vectors each: For expression of the GFP reporter cassettes in the context of P_{clag}-*cdpk1*, insertion was upstream of the *hdhfr* cassette in pSS367 (see above). For expression of the GFP reporter cassettes in the context of P_{cdpk1}-*cdpk1*, a vector similar to pSS367 was created, where the *clag* promoter was replaced with 2kb upstream sequence of *cdpk1*, to act as a *cdpk1* promoter. The GFP reporter cassettes were then inserted upstream of the *hdhfr* cassette in this vector. These cloning steps ultimately resulted in plasmids pSS378 (P_{gap50}-*gfp*/P_{cdpk1}-*cdpk1*), pSS379 (P_{mtip}-*gfp*/P_{cdpk1}-*cdpk1*), pSS380 (P_{gap50}-*gfp*/P_{clag}-*cdpk1*), and pSS381 (P_{mtip}-*gfp*/P_{clag}-*cdpk1*).

Protein and DNA blotting

For western blot analysis, parasite pellets were lysed in RIPA buffer (50 mM Tris, 150 mM NaCl, 0.1% SDS, 0.5% Triton X 100) for 20 min on ice. The insoluble pellet was removed by centrifugation (4°C, 15 min, 14,000rcf), and the supernatant was boiled for 10 min with 4x SDS-sample buffer before loading on a 4-10% BisTris polyacrylamide gel (Invitrogen). Proteins were blotted onto a PVDF membrane using the XCell II Blot module (Invitrogen) according to the manufacturer's instructions. Membranes were probed with anti-GFP rabbit polyclonal (Invitrogen), anti-PfMyoA rabbit polyclonal (Matuschewski et al., 2001), anti-PfGAP50, anti-PfGAP45, anti-PfMTIP (all rabbit polyclonals, Julian Rayner), anti-tubulin (Sigma, clone DM1A).

For Southern blots, genomic parasite DNA was isolated from mixed blood stages using phenol-chloroform extraction. After overnight restriction digest with EcoRI, samples were run on a 1% agarose gel and blotted onto a N+Hybond nylon membrane. For probing and detection, the DIG High Prime DNA Labeling and Detection Starter Kit II (Roche) was used according to the manufacturer's instructions. The *cdpk1* probe comprised the first 500 bp of *cdpk1* coding sequence.

Additional mass spectrometry methods

Peptides were desalted on-line using a micro-Precolumn cartridge (C18 Pepmap 100, LC Packings) and then separated using a 60 min RP gradient (4-32% acetonitrile/0.1% formic acid) on a BEH C18 analytical column (1.7 um, 75235 µm id x 10 cm, Waters). The mass spectrometer was operated in standard data dependent acquisition mode controlled by Xcalibur 2.1. The LTQ-Orbitrap Velos was operated with a cycle of one MS (in the Orbitrap) acquired at a resolution of 60,000 at m/z 400, with the top 10 most abundant multiply-charged (2+ and higher) ions in a given chromatographic window subjected to MS/MS fragmentation in the linear ion trap. FTMS target values of 1e6 and an ion trap MSn target values of 1e4 were used. Maximum FTMS scan accumulation times of was set at 500 ms and maximum ion trap MSn scan accumulation time was set to 100 ms. Dynamic exclusion was enabled with a repeat duration of 45 s with an exclusion list of 500 and exclusion duration of 30s. Acquisition was performed for 50 min for each gel band.

Silac labelled data was analysed using MaxQuant version 1.0.13.13 (Cox and Mann, 2008). and Mascot server 2.2 (Matrix Science) MaxQuant processed data was searched against a combined mouse and *Plasmodium berghei* protein sequence database using the following search parameters: trypsin with a maximum of 2 missed cleavages, 7 ppm for MS mass tolerance, 0.5 Da for MS/MS mass tolerance, with acetyl (Protein N-term) and oxidation (M) (as well as phospho S/T set as variable modifications) and carbamidomethyl (C) as a fixed modification. A protein FDR of 0.01 and a peptide FDR of 0.01 were used for identification level cut offs. Protein quantification was performed using razor and unique peptides and using only unmodified and carbamidomethylated peptides.

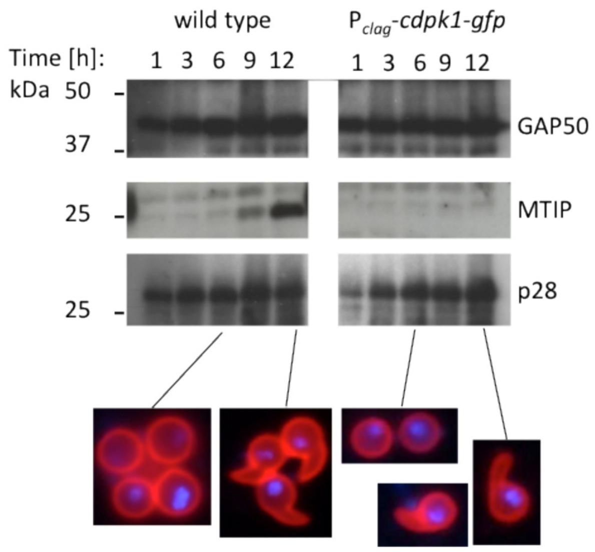

Figure S1. Kinetic Analysis of MTIP Expression in Wild Type and P_{clag}-*cdpk1-gfp* Ookinetes, Related to Figure 2. Western blot showing enhanced expression of MTIP in wild type between 9 -12 h after gametocyte activation, when retorts begin to form. Fluorescence micrographs show parasites from the same cultures used for protein analysis. GAP50 and p28 served as loading controls.

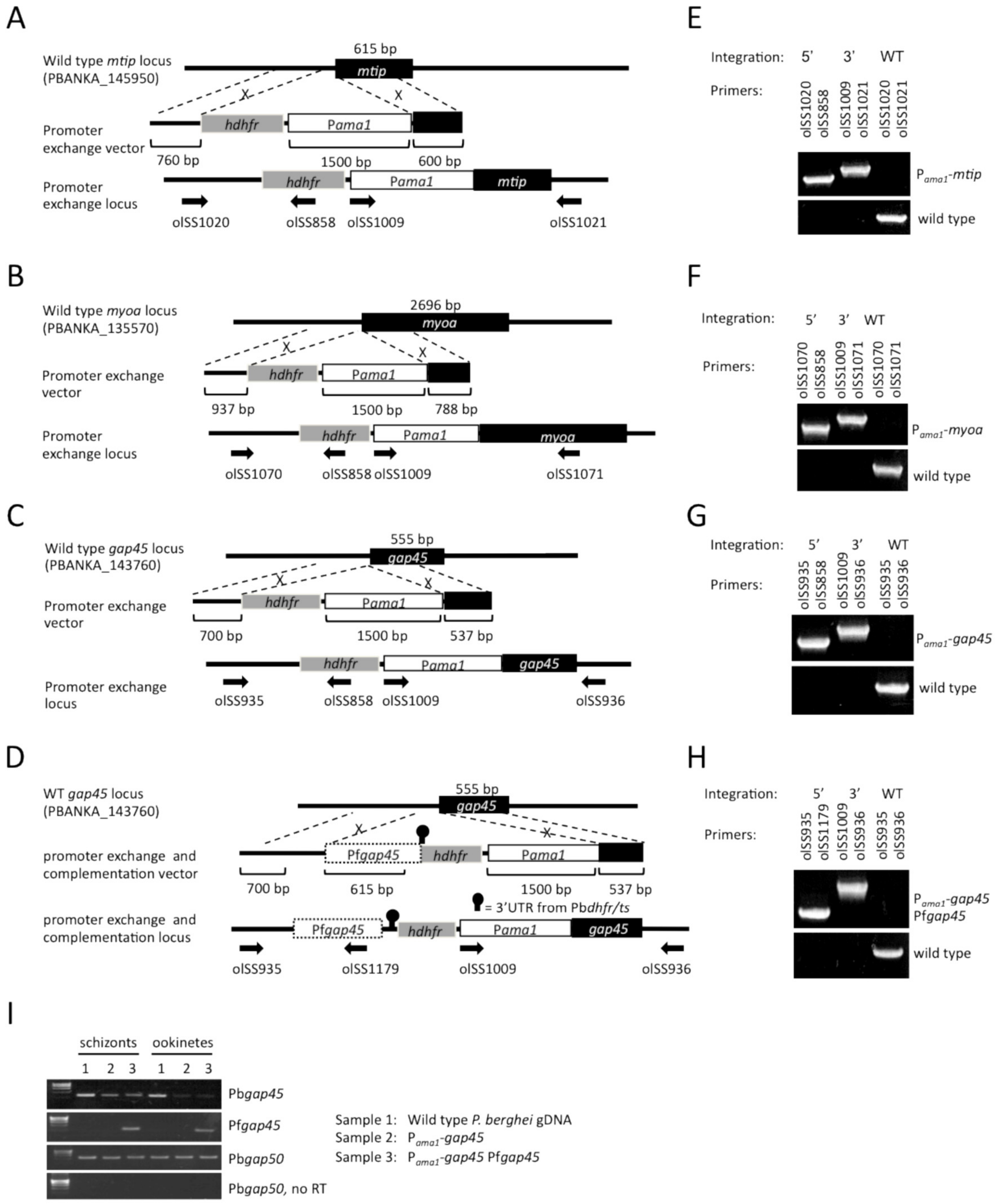

Figure S2. Promoter Exchange Mutants for the Motor Complex: Production and Genotyping, Related to Figure 3. A-C Schematics illustrating insertion of 1.5 kb upstream sequence from *ama1* into the endogenous loci *of mtip, myoa* and *gap45.* Oligonucleotides used for PCR genotyping are also shown. **D** Schematic illustrating simultaneous complementation of the *gap45* knockdown with *P. falciparum gap45.* **E-H** Agarose gel showing PCR products from genotyping reactions. **I** Impact of *ama1* promoter exchange and complementation on the presence of Pb*gap45* and Pf*gap45* transcripts in schizonts and ookinetes. An agarose gel shows products of an RT-PCR analysis on mRNA from ookinetes from 24hr *in vitro* cultures.

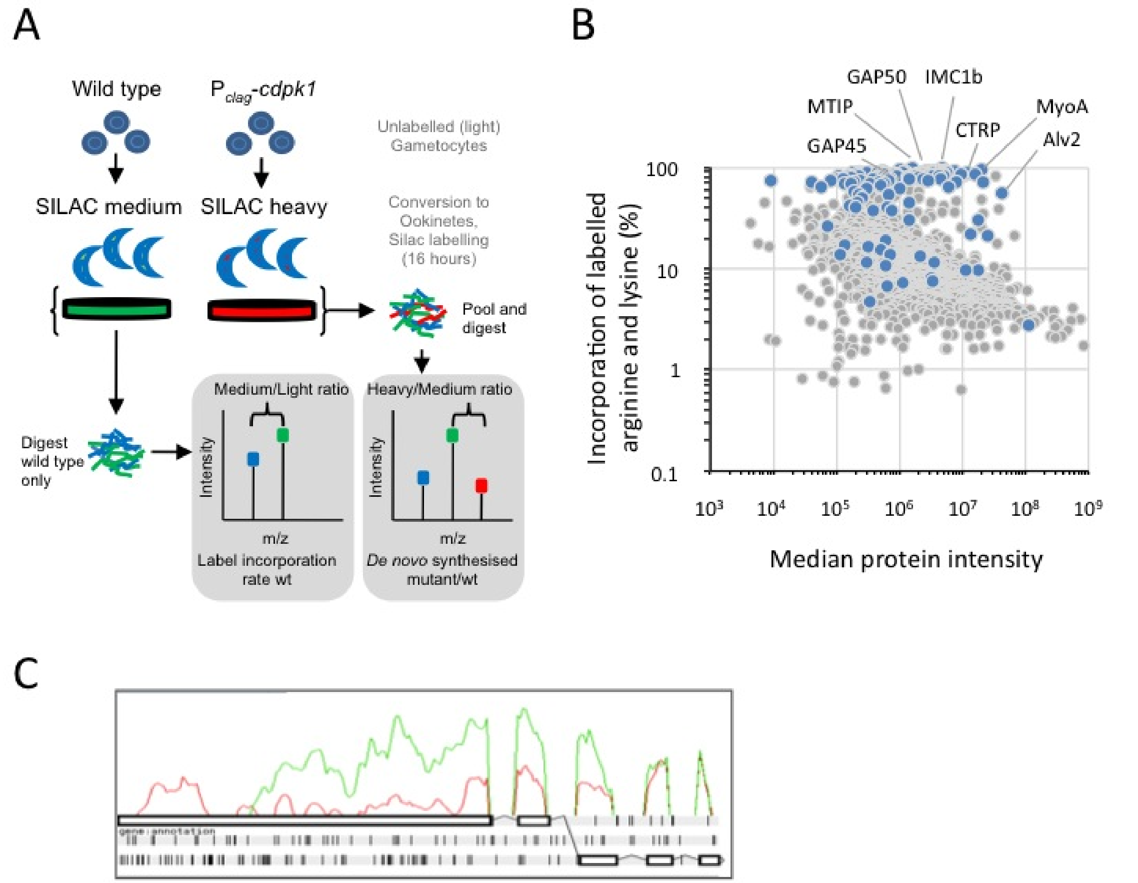

Figure S3. Proteome and Transcriptome Analysis of P_{clag}-*cdpk1* Ookinetes, Related to Figure 4. A. Work flow for pulse-chase SILAC labelling experiments with isotope labelled amino acids. Different types of experiments were performed to monitor *de novo* protein synthesis in wild type ookinetes and to quantify proteome changes between P_{clag}-*cdpk1* and wild type ookinetes. To quantify newly synthesized ookinete proteins, wild type SILAC labelled (medium) ookinetes were analysed by high resolution mass spectrometry in which peptides from newly synthesized proteins are 4/6 Daltons heavier than peptides from pre-existing proteins and the ratio of medium/light peptides is used to calculate the rate of newly synthesized proteins. In separate experiments, P_{clag}-*cdpk1* SILAC labelled (heavy) ookinetes were pooled with wild type (medium) ookinetes and analysed by mass spectrometry. In this case, peptides from P_{clag}-*cdpk1* proteins are 4 Daltons heavier than wild type medium labelled peptides and the heavy/medium ratio is a measure of the relative levels of proteins in P_{clag}-*cdpk1* and wild type ookinetes. **B.** Incorporation of isotope labelled amino acids during ookinete development plotted against protein abundance as reflected by summed intensities. Blue symbols show genes whose transcripts are dysregulated in a DOZI knock out mutant (Mair *et al*., 2006). **C.** Unexpected expression of a *cdpk1* fragment in the knock down mutant. RNA sequencing reads covered the complete *cdpk1* gene of wild type parasites (red), as expected. In the P_{clag}-*cdpk1-gfp* mutant, a 3' fragment of *cdpk1* was unexpectedly transcribed (green). Lack of N-terminal coverage shows that mRNA in the P_{clag}-*cdpk1-gfp* mutant originate from the c-terminal gene fragment that lacks an intact kinase domain and was created by the single cross over insertion of the GFP protein tag. The fragment appears to be transcribed and spliced despite the absence of a nominal promoter and 5' gene fragment encoding the kinase domain. CDPK1 derived peptides were present in the P_{clag}-*cdpk1-gfp* proteome (Table S1B), suggesting that the partial cDNA is translated into a polypeptide lacking a complete kinase domain.

Supplemental References

Cox, J., and Mann, M. (2008). MaxQuant enables high peptide identification rates, individualized p.p.b.-range mass accuracies and proteome-wide protein quantification. Nat Biotechnol *26*, 1367-1372.

Matuschewski, K., Mota, M.M., Pinder, J.C., Nussenzweig, V., and Kappe, S.H. (2001). Identification of the class XIV myosins Pb-MyoA and Py-MyoA and expression in Plasmodium sporozoites. Mol Biochem Parasitol *112*, 157-161.

van Spaendonk, R.M., Ramesar, J., van Wigcheren, A., Eling, W., Beetsma, A.L., van Gemert, G.J., Hooghof, J., Janse, C.J., and Waters, A.P. (2001). Functional equivalence of structurally distinct ribosomes in the malaria parasite, Plasmodium berghei. Journal of Biological Chemistry *276*, 22638-22647.
